# Genome analysis of *Streptococcus* spp. isolates from animals in pre-antibiotic era with respect to antibiotic susceptibility and virulence gene profiles

**DOI:** 10.1186/s13567-024-01302-0

**Published:** 2024-04-15

**Authors:** Ji-Yeon Hyeon, Junwon Kim, David H. Chung, Zeinab H. Helal, Robert Polkowski, Dong-Hun Lee, Guillermo R. Risatti

**Affiliations:** 1 College of Veterinary Medicine, Konkuk University, Seoul, Republic of Korea; 2 Department of Pathobiology and Veterinary Science, College of Agriculture, Health and Natural Resources, University of Connecticut, Storrs, CT USA; 3 Connecticut Veterinary Medical Diagnostic Laboratory, Department of Pathobiology and Veterinary Science, College of Agriculture, Health and Natural Resources, University of Connecticut, Storrs, CT USA

**Keywords:** *Streptococcus* spp., pre-antibiotic era, whole genome sequencing, antibiotic resistance, virulence profile

## Abstract

Lyophilized *Streptococcus* spp. isolates (*n* = 50) from animal samples submitted to the diagnostic laboratory at the University of Connecticut in the 1940s were revivified to investigate the genetic characteristics using whole-genome sequencing (WGS). The *Streptococcus* spp. isolates were identified as follows; *S. agalactiae* (*n* = 14), *S. dysgalactiae* subsp. *dysgalactiae* (*n* = 10)*, S. dysgalactiae* subsp. *equisimils* (*n* = 5)*, S. uberis* (*n* = 8), *S. pyogenes* (*n* = 7), *S. equi* subsp. *zooepidemicus* (*n* = 4), *S. oralis* (*n* = 1), and *S. pseudoporcinus* (*n* = 1)*.* We identified sequence types (ST) of *S. agalactiae*, *S. dysgalactiae*, *S. uberis*, *S. pyogenes*, and *S. equi* subsp. *zooepidemicus* and reported ten novel sequence types of those species. WGS analysis revealed that none of *Streptococcus* spp. carried antibiotic resistance genes. However, tetracycline resistance was observed in four out of 15 *S. dysgalactiae* isolates and in one out of four *S. equi* subsp. *zooepidemicus* isolate. This data highlights that antimicrobial resistance is pre-existed in nature before the use of antibiotics. The draft genome sequences of isolates from this study and 426 complete genome sequences of *Streptococcus* spp. downloaded from BV-BRC and NCBI GenBank database were analyzed for virulence gene profiles and phylogenetic relationships. Different *Streptococcus* species demonstrated distinct virulence gene profiles, with no time-related variations observed. Phylogenetic analysis revealed high genetic diversity of *Streptococcus* spp. isolates from the 1940s, and no clear spatio-temporal clustering patterns were observed among *Streptococcus* spp. analyzed in this study. This study provides an invaluable resource for studying the evolutionary aspects of antibiotic resistance acquisition and virulence in *Streptococcus* spp.

**Supplementary Information:**

The online version contains supplementary material available at 10.1186/s13567-024-01302-0.

## Introduction

Streptococci are Gram-positive bacteria that can be classified into the Lancefield group taxonomic system based on colony morphology, hemolysis, and serological specificity [1]. Many streptococci are non-pathogenic and belong to the commensal microbiota of humans and animals; however, some can cause severe diseases and health issues [1]. Several *Streptococcus* species can cause bovine mastitis (e.g., *S. uberis*, *S. agalactiae*, *S. dysgalactiae* subsp. *dysgalactiae*, and *S. canis*) and are responsible for major economic losses in the dairy industry [2, 3]. Various species of *Streptococcus,* such as *S. equi*, *S. suis*, *S. porcinus*, *S. oralis*, and *S. iniae* are associated with infections in pigs, horses, sheep, birds, aquatic mammals, and fish [4]*.*

*Streptococcus* spp. is typically sensitive to penicillin, which has been used as the drug of choice to combat gram-positive mastitis-causing organisms since 1945 [5, 6]. However, *Streptococcus* spp. quickly developed resistance to antibiotics, and the limited efficacy of mastitis control through the treatment of clinical cases was first noted by Murphy et al*.* in 1956 [7]. As a result, conventional antibiotic therapy often proves ineffective [5].

To improve our understanding of the emergence of antimicrobial resistance (AMR) and the evolution of bacterial pathogens, the AMR and genetic characteristics of historical isolates from before the widespread clinical use of antimicrobials, that is, the “pre-antibiotic” era, have been analyzed in previous studies, including those on *Klebsiella pneumoniae* [8], methicillin-resistant *Staphylococcus aureus* [9], *Salmonella enterica* serotype Typhimurium [10], *Neisseria gonorrhoeae* [11], *Vibrio cholerae* [12], and *Enterobacteriaceae* [13]. These studies demonstrated that a significant proportion of isolates from the pre-antibiotic era were resistant to antibiotics before their routine use, and the association between antibiotic use and selection of resistance determinants was not as direct as often presumed.

In this study, we sequenced the whole genomes of 50 lyophilized *Streptococcus* spp. isolates from clinical animal samples submitted to the diagnostic laboratory at the University of Connecticut in the pre-antibiotic era (1940s). We analyzed the phenotypic AMR, presence of AMR genes in the draft genomes, the sequence type (ST) using multi-locus sequence typing (MLST), phylogenetic relationships, and virulence gene profiles to examine the genetic characteristics of these historical isolates.

## Materials and methods

### Reviving lyophilized *Streptococcus* spp. isolates

A total of 50 lyophilized *Streptococcus* spp. cultures isolated from animal samples and stored by the Connecticut Veterinary Medical Diagnostic Laboratory (CVMDL), Department of Pathobiology and Veterinary Science, University of Connecticut, between 1941 and 1947 were revivified according to the Reviving Freeze-Dried Microorganisms Instructional Guide method published by the American Type Culture Collection (ATCC). Information on the isolates includes the isolation year and bacterial species which were indicated in the stock list but lacks other metadata such as host and disease information.

Briefly, lyophilized bacterial stocks were rehydrated and cultured in Tryptic Soy Broth medium (TSB) (Becton Dickinson, Franklin Lakes, NJ) for 24 h at 37 ℃. The cultured broth was streaked on blood agar plates, followed by incubation for an additional 24 h at 37 ℃. Next, colonies from blood agar plates (a colony from each plate) were cultured in TSB for 24 h at 37 ℃. The cultures were stored in the Cryocare Bead Storage system (Key Scientific Product, Stamford, Texas) at −80 ℃ until ready for analysis.

### Antimicrobial susceptibility testing

The antimicrobial susceptibility of *Streptococcus* spp. isolates was determined using a Sensititre^™^ Streptococcus STP6F AST Plate (Thermo Fisher Scientific, Waltham, MA) which is a colorimetric microdilution test consisting of the following 20 antimicrobials: moxifloxacin, levofloxacin, tetracycline, cefuroxime, ceftriaxone, cefotaxime, daptomycin, chloramphenicol, penicillin, meropenem, ertapenem, amoxicillin/clavulanic acid 2:1 ratio, linezolid, clindamycin, cefepime, tigecycline, azithromycin, erythromycin, trimethoprim/sulfamethoxazole, and vancomycin. Briefly, bacterial colonies were suspended in sterile distilled water to approximate the 0.5 McFarland turbidity standard. Next, 100 µL of the bacterial suspension was transferred into 5 mL of Sensititre^™^ Mueller Hinton broth with lysed horse blood, and 100 µL of the inoculum was inoculated into each well of a Sensititre^™^ Streptococcus species STP6F susceptibility plate. After 24 h of incubation at 37 ℃, the minimum inhibitory concentration (MIC) was determined using a BIOMIC V3 Microbiology system (Giles Scientific Inc., Santa Barbara, CA) according to the manufacturer’s instructions. The AMR of the isolates was determined according to the concentrations of each drug range and interpretive criteria in the instruction of M100 of the Clinical and Laboratory Standards Institute (CLSI) [14].

### Whole genome sequencing (WGS)

For WGS, genomic DNA was extracted from pure cultures of *Streptococcus* spp. using the DNeasy Blood and Tissue kit (Qiagen, Valencia, CA, USA) according to the manufacturer’s instructions. Sample DNA concentrations were determined using a Qubit dsDNA HS assay kit (Invitrogen, Carlsbad, CA, USA), and DNA samples were diluted to 0.2 ng/µL. Sample libraries were prepared using the Illumina Nextera XT DNA Library Prep Kit (Illumina, San Diego, CA, USA), followed by dilution to a concentration of 2 nM; the concentration of libraries was measured using the Qubit dsDNA HS assay kit. Samples were sequenced using a MiSeq Reagent Kit V2 (500 cycle) cartridge (Illumina) after loading 600 µL of the 10 pM pooled libraries.

### Species identification

The BIOLOG MicroLog3 Microbial Identification System (Biolog, Hayward, CA, USA) was used to identify the species of all isolates. For the confirmation of bacterial species identification, we analyzed the genome sequences of 16S rRNA region. The 16S rRNA region in the assembled contigs of the isolates was predicted using bacterial ribosomal RNA predictor barnap (Galaxy Version 1.2.1) and extracted manually. For each 16S rRNA sequence, the nearest-neighbor species with > 99% identity were searched using the Basic Local Alignment Search Tool (BLAST) on the National Center for Biotechnology Information (NCBI) database to identify the species of each isolate with the default parameters.

### Genomic characterization

Raw reads were de novo assembled using the SPAdes algorithm [15] at the BV-BRC online server. The assembled contigs with a coverage of less than 5 × and sizes below 300 bases were removed. The presence of acquired antimicrobial resistance genes was determined using ResFinder 3.2 with settings for other species, a threshold of 90%, and a minimum length of 60% with raw sequencing reads [16]. Plasmids were detected using PlasmidFinder 2.1 [17], a web-based tool for in silico detection and characterization of plasmid sequences based on BLAST searches against plasmid replicon genes with the assembled contigs of gram-positive bacteria. MLST 2.0 (Multi-Locus Sequence Typing) [18] was used to determine the STs of the predicted species using the assembled contigs. The virulence genes of the isolates used for the phylogenetic analysis including our isolates (Additional file 1) were analyzed using ABRicate (Version 1.0.1) against virulence factor database (VFDB) [19], with a 80% sequence identity and a 80% coverage.

### Phylogenetic analysis

All available complete genome sequences of *S. agalactiae* (*n* = 185), *S. dysgalactiae* (*n* = 23), *S. equi* subsp. *zooepidemicus* (*n* = 25)*,* and *S. pyogenes* (*n* = 193) with collection year information were downloaded from BV-BRC and NCBI GenBank database to investigate the genetic relationships between the isolates. The species which the number of complete genomes in the NCBI GenBank database was less than 20 as of February 21, 2024, were excluded from the phylogenetic analysis; *S. oralis* (*n* = 19), *S. uberis* (*n* = 4), and *S. pseudoporcinus* (*n* = 3). The information of the genome sequences used for the analysis is listed in Additional file 1. Whole genome SNPs were identified using kSNP4 [20] which employs an alignment-free approach for SNP identification. The SNPs-based ML tree was generated using FastTree [21], which was automatically applied in the kSNP4 pipeline.

## Results

### Genomic characteristics

The de novo assembly results and genomic characteristics of the isolates are shown in Additional file 2. The average depth of coverage ranged from 40.8 to 187.1, the number of contigs from 12 to 167, and the N50 from 32 972 to 1 034 038. The average number of protein coding sequences (CDS) of each species ranged from 1795 (*S. pyogenes*) to 2301 (*S. equi* subsp. *Zooepidemicus*), the rRNA was from 4 to 5*,* and the tRNA was from 39 (*S. pyogenes*) to 51 (*S. uberis*) (Additional file 2).

In this study, the average GC content for each species was 35% for the *S. agalactiae* isolates, 39% for the *S. dysgalactiae* isolates, 36% for the *S. uberis* isolates, 38% for the *S. pyogenes* isolates, 41% for the *S. equi* subsp*. zooepidemicus* isolates, 40.9% for the *S. oralis* isolate, and 37.3% for the *S. pseudoporcinus* isolate (Additional file 2).

### Species identification, sequence type and plasmid of *Streptococcus* spp.

The *Streptococcus* spp. isolates (*n* = 50) included 14 *S. agalactiae*, 10 *S. dysgalactiae* subsp. *dysgalactiae,* five *S. dysgalactiae* subsp. *equisimils,* eight *S. uberis*, seven *S. pyogenes*, four *S. equi* subsp. *zooepidemicus*, one *S. oralis*, and one *S. pseudoporcinus* (Table 1).

**Table 1:** **Antibiotic resistance genotypes and phenotypes, sequence types (ST), and plasmids of** ***Streptococcus* spp. isolates from 1940s analyzed in this study (*n* = 50)**

Isolates	Isolation year	Sequence Type	Genotype	Phenotype (MIC, μg/mL)^b^	Plasmid
*S. agalactiae*					
16	1941	356	-	S	
39	1941	61	-	S	
090R	1941	25	-	S	
B1006	1947	61	-	S	
B2142	1941	23	-	S	
B2151	1941	23	-	S	
G2	1941	61	-	S	pA996 and pSSU1
G19	1947	61	-	S	pA996 and pSSU1
G42	1948	61	-	S	
H36B	1941	6	-	S	
N49	1941	2225 ^a^	-	S	
S101	1941	23	-	S	
S102	1948	64	-	S	
S104	1948	23	-	S	
*S. dysgalactiae* subsp.* dysgalactiae*					
21	1941	298	-	S	
36	1941	532	-	Tetracycline R (8)	
41	1941	308	-	Tetracycline R(8)	
43	1941	531	-	Tetracycline I (4)	
45	1941	308	-	Tetracycline I (4)	
46	1941	298	-	S	
B2198	1941	531	-	Tetracycline I (4)	
B2200	1941	531	-	Tetracycline I (4)	
B2263	1941	531	-	S	
B2273	1941	531	-	Tetracycline R (8)	
*S. dysgalactiae* subsp.* equisimilis*					
18	1941	3	-	S	
19	1941	275	-	S	
29	1941	641	-	S	
30	1941	84	-	S	
34	1941	723 ^a^	-	Tetracycline R (8)	
*S. uberis*					
17	1941	472	-	S	
B2160	1947	1802 ^a^	-	S	pA996
B2254	1941	1815 ^a^	-	S	
B2258	1941	1815 ^a^	-	S	
B2165	1941	1801 ^a^	-	S	
B2139	1941	1817 ^a^	-	S	
B2141	1941	1818 ^a^	-	S	
U84	1947	1804 ^a^	-	S	
*S. pyogenes*					
5	1941	38	-	S	
6	1941	28	-	S	
7	1941	1278	-	S	
9	1941	28	-	S	
12	1941	26	-	S	
14	1941	28	-	S	
15	1941	120	-	S	
*S. equi* subsp. *zooepidemicus*					
24	1941	529 ^a^	-	Tetracycline I (4)	
37	1941	182	-	Tetracycline I (4), Clindamycin I (0.5)	
38	1941	530 ^a^	-	Clindamycin I (0.5)	
40	1941	27	-	Tetracycline R (8), Clindamycin I (0.5)	
*S. oralis*					
11	1941	–	-	S	
*S. pseudoporcinus*					
28	1941	–	-	S	

^a^Novel ST

^b^S-sensitive; I-intermediate; R-resistant

We identified STs of *S. agalactiae*, *S. dysgalactiae*, *S. uberis*, *S. pyogenes*, and *S. equi* subsp. *zooepidemicus*, which are available in MLST 2.0. (Table 1). The most frequent STs were ST 61 in *S. agalactiae* isolates (5 out of 14), ST 531 in *S. dysgalactiae* isolates (5 out of 15), and ST28 in *S. pyogenes* isolates (3 out of 7). In this study, we reported the novel sequence types of one *S. agalactiae* isolates (ST 2225), one *S. dysgalactiae* isolates (ST 723), seven *S. uberis* isolates (ST 1801, 1802, 1804, 1815, 1817, and 1818), and two *S. equi* subsp*. zooepidemicus* (ST 529 and 530) isolates (Table 1 and Additional file 3).

Plasmid detection via the PlasmidFinder 2.1 revealed that among the 50 *Streptococcus* spp. isolates, two *S. agalactiae* (G2 and G19) carried two plasmids, pA996 and pSSU1, and one *S. uberis* isolate carried the pA996 plasmid (Table 1). It should be noted that plasmid fragments without replicons may have been missed in this analysis since the PlasmidFinder 2.1 identifies plasmids based on replicon sequences.

### Antibiotic resistance of *Streptococcus* spp.

The presence of antimicrobial resistance genes and phenotypic antimicrobial susceptibility testing of *Streptococcus* spp. isolates are shown in Table 1. Antimicrobial resistance genes were not found in the *Streptococcus* spp.isolates. All of *S. uberi, S. pyogenes*, *S. oralis*, and *S. pseudoporcinus* isolates were susceptible to all antibiotics tested. However, phenotypic resistance to tetracycline was observed in three out of ten *S. dysgalactiae* subsp. *dysgalactiae* isolates, one out of five *S. dysgalactiae* subsp. *equimilis* isolate*,* and one out of four *S. equi* subsp. *zooepidemicus* isolate. Four of ten *S. dysgalactiae* subsp. *dysgalactiae* isolates and two out of four *S. equi* subsp. *zooepidemicus* isolates showed intermediate resistance to tetracycline. In addition, three out of four *S. equi* subsp. *zooepidemicus* isolates showed intermediate resistance to clindamycin.

### Virulence gene profile

The virulence gene profiles of 50 *Streptococcus* spp. isolates sequenced in this study were analyzed and compared with those of 426 complete genome sequences of *Streptococcus* spp. downloaded from BV-BRC and NCBI GenBank database (Tables 2, 3, 4, 5 and Additional file 4).

**Table 2:** **Virulence gene profiles of** ***S. agalactiae*** **isolates from 1940s analyzed in this study (*n*** **= 14)**

Strains	Adherence										Antiphagocytosis													Exoenzyme	Immune evasion	Toxin											
	*fbsB*	*gbs0628*	*gbs0630*	*gbs0631*	*gbs0632*	*lmb*	*pilA*	*pilB*	*pilC*	*srtC4*	*cpsA*	*cpsB*	*cpsC*	*cpsD*	*cpsE*	*cpsF*	*cpsG*	*cpsK*	*cpsL*	*neuA*	*neuB*	*neuC*	*neuD*	*hylB*	*scpB*	*acpC*	*cfa/cfb*	*cylA*	*cylB*	*cylD*	*cylF*	*cylG*	*cylI*	*cylJ*	*cylK*	*cylX*	*cylZ*
*S.agalactiae*_B090R	+	+	+	+	+	+	+		+	+	+	+	+	+	+	+		+	+	+	+	+	+	+	+	+	+	+	+	+	+	+	+	+	+	+	+
*S.agalactiae*_N49			+	+	+						+	+	+	+	+	+	+		+	+	+	+	+	+		+	+	+	+	+	+	+	+	+	+	+	+
*S.agalactiae*_H36B						+	+		+	+	+	+	+	+	+	+			+	+	+	+	+	+	+	+	+	+	+	+	+	+	+	+	+	+	+
*S.agalactiae*_S101	+	+	+	+	+		+		+	+	+	+	+	+	+	+		+	+	+	+	+	+	+		+	+	+	+	+	+	+	+	+	+	+	+
*S.agalactiae*_B2142	+	+	+	+	+	+	+	+	+	+	+	+	+	+	+	+		+	+	+	+	+	+	+	+	+	+	+	+	+	+	+	+	+	+	+	+
*S.agalactiae*_B2151	+	+	+	+	+	+	+	+	+	+	+	+	+	+	+	+		+	+	+	+	+	+	+	+	+	+	+	+	+	+	+	+	+	+	+	+
*S.agalactiae*_G2											+	+	+	+	+	+	+		+	+	+	+	+	+			+										
*S.agalactiae*_39											+	+	+	+	+	+		+	+	+	+	+	+			+	+	+	+	+	+	+	+	+	+	+	+
*S.agalactiae*_16											+	+	+	+	+	+	+		+	+	+	+	+	+			+			+						+	
*S.agalactiae*_B1006											+	+	+	+	+	+			+	+	+	+	+	+			+										
*S.agalactiae*_G19											+	+	+	+	+	+	+		+	+	+	+	+	+			+										
*S.agalactiae*_S104	+	+	+	+	+		+		+	+	+	+	+	+	+	+		+	+	+	+	+	+	+		+	+	+	+	+	+	+	+	+	+	+	+
*S.agalactiae*_G42											+	+	+	+	+	+			+	+	+	+	+	+			+										
*S.agalactiae*_S102											+	+	+	+	+	+	+		+	+	+	+	+	+		+	+	+	+	+	+	+	+	+		+	+

**Table 3:** **Virulence gene profiles of** ***S. dysgalactiae*** **isolates from 1940s analyzed in this study (*n* = 15)**

Strains	Adherence					Antiphagocytosis	Exoenzyme			Immune evasion			Toxin					
	*fbp54*	*gbs0630*	*gbs0631*	*gbs0632*	*lmb*	*hasC*	*mf2*	*mf3*	*sda*	*scpA*	*scpB*	*ska*	*slo*	*spec*	*speg*	*spek*	*spel*	*spem*
*S. dysgalactiae subsp. equisimilis_*18	+	+	+	+	+	+					+	+	+		+			
*S. dysgalactiae subsp. equisimilis*_19	+				+	+				+		+	+					
*S. dysgalactiae subsp. equisimilis*_29	+	+	+	+	+	+				+		+	+					
*S. dysgalactiae subsp. equisimilis*_30	+	+	+	+	+	+				+		+	+					
*S. dysgalactiae subsp. equisimilis*_34	+					+												
*S. dysgalactiae* subsp. *dysgalactiae*_21	+					+		+								+	+	
*S. dysgalactiae* subsp. *dysgalactiae*_B2263	+					+												+
*S. dysgalactiae* subsp. *dysgalactiae*_B2273	+					+												+
*S. dysgalactiae* subsp. *dysgalactiae*_36	+					+		+								+	+	+
*S. dysgalactiae* subsp. *dysgalactiae*_41	+					+		+	+							+	+	
*S. dysgalactiae* subsp. *dysgalactiae*_43	+					+												+
*S. dysgalactiae* subsp. *dysgalactiae*_45	+					+		+	+							+	+	
*S. dysgalactiae* subsp. *dysgalactiae*_46	+					+												+
*S. dysgalactiae* subsp. *dysgalactiae*_B2198	+					+												+
*S. dysgalactiae* subsp. *dysgalactiae_*B2200	+					+												+

**Table 4:** **Virulence gene profiles of *S. pyogenes* isolates from 1940s analyzed in this study (*n* = 7)**

Strains	Adherence								Anti-proteolysis							Exoenzyme						Immune evasion		Toxin												
	*cpa*	*fbaA*	*fbaB*	*fctA*	*fctB*	*lepA*	*lmb*	*srtC1*	*grab*	*emm*	*hasA*	*hasB*	*hasC*	*ideS/mac*	*sic*	*hylP*	*mf/spd*	*mf2*	*mf3*	*sda*	*speB*	*scpA*	*ska*	*fbp54*	*slo*	*smeZ*	*spea*	*spec*	*speg*	*speh*	*spei*	*spej*	*spek*	*spel*	*spem*	*ssa*
*S. pyogenes*_12							+				+	+	+	+		+	+	+			+	+	+	+	+	+		+	+	+	+					
*S. pyogenes*_14	+			+	+	+	+	+	+		+	+	+	+				+	+			+	+	+	+	+		+	+	+	+	+				
*S. pyogenes*_15	+	+	+				+				+	+	+	+		+	+		+		+	+	+	+	+	+	+		+	+						
*S. pyogenes_*5							+							+		+	+	+	+		+	+	+	+	+	+		+								+
*S. pyogenes*_6	+			+	+	+	+	+	+	+	+	+	+	+	+		+	+	+		+	+	+	+	+	+		+	+	+	+	+				
*S. pyogenes*_7	+						+				+	+	+	+			+				+	+	+	+	+	+			+							
*S. pyogenes*_9	+			+	+	+	+	+	+	+	+	+	+	+	+		+	+	+		+	+	+	+	+	+		+	+	+	+	+

**Table 5:** **Virulence gene profiles of** ***S. equi*** **subsp.** ***zooepidemicus*** **(*n* = 4),** ***S. oralis* (*n*** **= 1), and *S. pseudoporcinus* (*n* = 1) isolates from 1940s analyzed in this study (*n* = 6)**

Strains	Adherence	Manganese uptake	Exoenzyme	
	pavA	psaA	mf2	speB
*S. equi*_subsp. *zooepidemicus*_24				
*S. equi*_subsp. *zooepidemicus*_37			+	
*S. equi*_subsp. *zooepidemicus*_38				
*S. equi*_subsp. *zooepidemicus*_40			+	
*S. oralis*_11	+	+		
*S. pseudoporcinus*_28				+

The virulence genes were classified into eight categories based on their function: adherence, anti-proteolysis, antiphagocytosis, exoenzymes, immune evasion, manganese uptake, stress proteins, and toxins (Additional file 4). Different *Streptococcus* species demonstrated distinct virulence gene profiles, and no time-related variations were observed in the virulence gene profile across all analyzed *Streptococcus* species (Additional file 4). All 14 *S. agalactiae* isolates sequenced in this study carried the genes related to antiphagocytosis (*cpsA-F*, *cpsL*, and *neuA-D*) and toxins (*cfa/cfb*) (Table 2). Among these genes, *cfa/cfb, cpsL,* and *neuB-D* genes were detected in all *S. agalactiae* complete genome sequences analyzed in this study (Additional file 4). All 38 genome sequences of *S. dysgalactiae* including isolates from this study harbored *fbp54* (adherence) and *hasC* genes (anti-proteolysis) (Table 3 and Additional file 4). All 200 *S. pyogenes* genome sequences carried *lmb* (adherence), *ideS/mac* (antiphagocytosis)*, fbp54* (toxin)*, ska* (toxin)*,* and *slo* (toxin) (Additional file 4), and all our *S. pyogenes* isolates (*n* = 7) additionally harbored s*cpA* (immune invasion) and *SmeZ* (toxin) (Table 4). Two virulence genes encoding exozyme, *hylP* and *mf2,* were observed in all analyzed *S. equi* subsp. *zooepidemicus*, and two of four *S. equi* subsp. *zooepidemicus* isolates from this study harbored only *mf2* gene (Table 5 and Additional file 4)*. S. pseudoporcinus* isolates carried *speB* genes encoding exozyme, while *S. oralis* isolates harbored the *pavA* (adherence) and *psaA* (magnese uptake) genes (Table 5)*.* Lastly, no virulence gene in VFDB was detected in our *S. uberis* isolates (Additional file 4).

### Phylogenetic analysis

The genome sequences of *S. agalactiae* (*n* = 199), *S. dysgalactiae* (*n* = 38), *S. pyogenes* (*n* = 200)*,* and *S. equi* subsp. *zooepidemicus* (*n* = 29) including our isolates were analyzed to investigate the genetic relationships using SNP analysis. The phylogenetic trees and the heatmaps of four different species are shown in Additional files 5, 6, 7 and 8.

The host species of 185 *S. agalactiae* sequences downloaded from databases were humans (*n* = 81), aquatic animals (*n* = 81), cows (*n* = 22), a dog (*n* = 1), and unknown (*n* = 11). *S. agalactiae* from aquatic animals formed two distinct clusters which are compressed in phylogeny (Additional file 5). The phylogenetic analysis revealed that our isolates were divided into four groups (Additional file 5). *S. agalactiae* N49 and H36B were grouped with isolates from cows in 1970 and 1954, respectively, exhibiting high sequence identity (82.6% and 87.2%, respectively). Five isolates (S101, S104, B090R, B2142, and B2151) demonstrated an average identity of 89.6% with two isolates, one from a cow in 1977 and another from a human in 2011. Seven isolates (B1006, G42, G19, G2, S102, 39, and 16) were grouped with two isolates from cows in 1954 and 1964 showing average 68.2% identity.

The sequences of *S. dysgalactiae* were divided into two subspecies, *S. dysgalactiae* subsp. *equisimillis* and *S. dysgalactiae* subsp. *dysgalactiae* (Additional file 6). Four *S. dysgalactiae* subsp. *equisimillis* isolates from this study (30, 29, 19, and 18) clustered with isolates from humans between 1953 and 2018, and three of them showed an average 78.3% identity with an isolate from a human in 1953. One isolate (34) was grouped with two isolates from a cow and a rhinoceros, exhibiting an average 58.8% identity. All *S. dysgalactiae* subsp. *dysgalactiae* isolates from this study clustered with an isolate from a cow in 2020, exhibited an average 76.0% identity.

*S. pyogenes* sequences available in the databases were from human isolates (*n* = 184) and unknown (*n* = 9). The *S. pyogenes* isolates of this study were grouped into five clusters (Additional file 7). The isolates (6, 14, and 9) were grouped with isolates from humans in 1950–2019, exhibiting an average 95.3% identity. *S. pyogenes* isolates 12 and 5 showed high identity with historical strains isolated in 1927 (90.6%) and 1950 (98.3%), respectively. *S. pyogenes* isolate 15 also exhibited high identity with human strains isolated between 1997 and 2016 (97.4%), and *S. pyogenes* isolate 7 were clustered with human strains isolated between 2009 and 2015 (84.6%). For *S. equi* subsp*. zooepidemicus*, four isolates of this study clustered into three different groups (Additional file 8). The clusters containing *S. equi* subsp*. zooepidemicus* isolates 37 and 38 exhibited average 52.1% and 54.0% identity and *S. equi* subsp*. zooepidemicus* isolates 40 and 24 clustered with an isolate from a cow with an average identity of 45.5%.

## Discussion

*Streptococcus* spp. have been identified using classical phenotypic microbiological procedures [22]. However, previous studies have reported the limited discriminatory power of these methods for *Streptococcus* spp. [22–25], and the 16S rRNA sequence started to be used as a reference for species identification [3, 22, 25]. Therefore, in the present study, we determined the species using a BLASTn search of the 16S rRNA sequences. One of the limitations of this study is the lack of information regarding the isolates, such as host and disease. Therefore, we assumed the host of each isolate based on the prevalence of *Streptococcus* spp. in different animals as reported in the previous studies. *S. agalactiae*, *S. dysgalactiae*, and *S. uberis* are the main species involved in clinical and subclinical bovine mastitis [1, 3, 5, 26]. A few bovine mastitis cases caused by *S. pyogenes* were reported between 1930 and 1940 [27, 28]. *S. equi* subsp. *zooepidemicus* is an opportunistic pathogen in both humans and a broad range of animal species, including horses, dogs, and pigs [29]. *S. oralis*, a member of the mitis group of streptococci, has been isolated from milk samples from women [30] and lactating cows [31]. *S. pseudoporcinus* was initially thought to be *S. porcinus*, which was first isolated frompigs in 1937 [32].

All *S. agalactiae* isolates of this study carried the *mre(A)* gene (data not shown), which is known to probably reside in *S. agalactiae* and may encode a metabolic function [33]. The *mre(A)* gene, which encodes a flavokinase, was discovered in a unique strain of *S. agalactiae* COH31 γ/δ as a macrolide efflux gene by Clancy et al*.* [34], and cumulative data suggested that the *mreA* gene was located on the chromosome of *S. agalactiae* COH31 γ/δ [33]. This is supported by our finding that all *S. agalactiae* isolates in this study carried the *mre(A)* gene with an erythromycin-sensitive phenotype. The *mre(A)* gene was found in all *S. agalactiae* isolates analyzed with either erythromycin-resistant or erythromycin-sensitive phenotypes in previous studies [33, 35, 36], indicating its ubiquity in this bacterial species. In this study, phenotypic resistance to tetracycline and intermediate resistance to clindamycin were observed in the *S. dysgalactiae* subsp. *dysgalactiae* isolates, *S. dysgalactiae* subsp. *equimilis* isolate*,* and *S. equi* subsp. *zooepidemicus* isolate, while resistance genes were not found in the *Streptococcus* spp. isolates. Tetracycline resistance in *S. agalactiae, S. dysgalactiae*, and *S. equi* subsp*. zooepidemicus* has been detected by several resistance monitoring programs in previous studies [29, 37], and AMR in *Streptococcus* spp. varies greatly depending on the streptococcal species, geographical location, study design (sampling size, scheme, and method for resistance determination), and literature source [1]. A poor correlation between tetracycline-resistant phenotypes and resistance genes has been reported previously [38–40]. In the previous study [40], six of 18 tetracycline resistant *S. dysgalactiae* subsp. *dysgalactiae* isolates did not carry the *tet* genes. In addition, in Tian et al.’s study on 64 *Streptococcus* isolates from mastitic milk samples in China [38], the average consistency between resistant phenotypes and resistance genes was 35.87%, and the consistency rate for tetracycline was 50%.

The phenotypic and genotypic AMR of bacterial pathogens from the pre-antibiotic era have been reported in previous studies, such as the Murray Collection of the pre-antibiotic era *Enterobacteriaceae* strains carrying antibiotic resistance genes [13], *Proteus* spp. resistant to tetracycline [41], *Klebsiella* resistant to ampicillin [8], *Escherichia* spp. resistant to both ampicillin and kanamycin [42], and *Vibrio cholerae* strains harboring functional β-lactamase antibiotic resistance genes [12]. In addition, metagenomic studies on ancient human guts from the pre-antibiotic era have been reported [43–45]. These investigations on the gut microbiome of pre-Columbian Andean [44], pre-Inca/Inca, and Italian nobility mummies [43, 45] revealed the presence of genes associated with beta-lactamases, penicillin-binding proteins, resistance to fosfomycin, chloramphenicol, aminoglycosides, macrolides, sulfa, quinolones, tetracycline, and vancomycin, as well as multi-drug transporters. This suggests that resistance may not necessarily be associated solely with the selective pressure of antibiotics.

Moreover, the studies propose that antibiotic resistance might have an environmental origin, indicating that a higher exposure to the environment could lead to a greater acquisition of antibiotic-resistance genes [43, 45]. Additionally, it has been hypothesized that antibiotic resistance in pathogens likely originated in non-pathogenic bacteria, possibly those originating from the soil [43, 45]. Contrastingly, our study has revealed that *Streptococcus* spp. isolates from animal origins during the pre-antibiotic era did not carry antibiotic resistance genes. This disparity in findings appears to be attributed to the differing origins (animal vs. human) and pathogenicity of the bacteria.

Several studies where PCR was used to screen for virulence genes have reported differences in the detection of the virulence factors of *Streptococcus* spp. from different sources, such as *S. agalactiae* strains from human and bovine sources [46, 47]. However, literature reports on the virulence gene profiles of *Streptococcus* spp. isolates from animals using WGS are scarce [39, 48]. In this study, we compared the virulence gene profiles of *Streptococcus* spp. isolates from 1940s with those of 426 complete genome sequences of *Streptococcus* spp. obtained from diverse hosts and different years to investigate potential time-related variations and evolutionary trends. The results revealed conserved virulence gene profiles among different *Streptococcus* species and no time-related variations in the virulence gene profile in analyzed *Streptococcus* species. This comprehensive approach provides insight into the diverse virulence gene pattern shaping the framework of *Streptococcus* spp. pathogenesis. A discrepancy in reporting of virulence gene prevalence was observed among different previous studies, which can be explained by the difference in the origin of the isolates as well as other factors [1, 5, 38, 46–50]. In addition, a limitation of VFDB is its primary focus on data from human pathogens, potentially overlooking virulence genes for animal pathogens. This raises concerns regarding the understanding of virulence gene datasets of *Streptococcus* spp. infecting animals, such as *S. oralis*, *S. uberis*, and *S. pseudoporcinus.* Therefore, further WGS analysis of *Streptococcus* spp. isolates from animals is essential to update the database and better understand the evolution of virulence genes in *Streptococcus* spp. from diverse host species.

In this study, all available complete genome sequences of *S. agalactiae*, *S. dysgalactiae*, *S. equi* subsp. *zooepidemicus*, and *S. pyogenes* with collection year information were downloaded from databases to investigate the genetic relationships with our isolates using SNP analysis. Phylogenetic analysis revealed high genetic diversity of *Streptococcus* spp. isolates from the 1940s, and no clear spatio-temporal clustering patterns were observed among *Streptococcus* spp. analyzed in this study. *S. agalactiae* isolates and *S. dysgalactiae* subsp. *dysgalactiae* isolates of this study exhibited high genetic similarity with the isolates from cows, suggesting a potential host-specific association. *S. dysgalactiae* subsp. *equismillis* and *S. pyogenes* displayed high genetic identity (> 90%) with both historical and contemporary human isolates, suggesting their persistence and adaptability within human populations overtime. However, the scarcity of sequence data for these species from animals constrained genetic analysis with animal isolates in this study. Further research with a wider range of animal isolates is needed to better understand genetic diversity and evolution of these subspecies.

This study reports on the antibiotic resistance, sequence type, phylogenetic relationships, and virulence gene profiles of lyophilized *Streptococcus* spp. isolated from animals in the 1940s, the pre-antibiotic era, using WGS analysis. This study provides an invaluable resource for further investigation of the evolutionary aspects of antibiotic resistance acquisition and adaptation of bacterial strains.

### Supplementary Information

**Additional file 1. The information of the complete genomes of** ***S. agalactiae *(*n *= 185),** ***S. dysgalactiae *(*n *= 23), *S. pyogenes*** **(*n *= 193), and *S. equi* subsp.* zooepidemicus *(*n *= 25)*,* used for phylogenetic analysis (*n *= 427).**

**Additional file 2. Genome characteristics and the de novo assembly results for the 50 *Streptococcus*** **spp. sequenced in this study.**

**Additional file 3. The MLST results of the *Streptococcus* spp. isolates with the novel STs.**

**Additional file 4. Virulence gene profiles the complete genome sequences of *Streptococcus*** **spp. used for phylogenetic analysis and 50 *Streptococcus* spp. isolates sequenced in this study (highlighted in red).**

**Additional file 5. Phylogenetic analysis of *S. agalactiae *(*n *= 199) including our isolates (highlighted in red) using SNP analysis. The phylogeny was rooted at midpoint. The scale bars show the number of substitutions per site. The numerical values represent 1000 bootstrap replicate values expressed as a percentage. Subtrees including the *S. agalactiae *from aquatic animals were compressed to better visualize the genetic relationships. The colors in the heat map represent the levels of identity (%) between isolates, with white indicating the lowest and green indicating the highest.**

**Additional file 6. Phylogenetic analysis of** ***S. dysgalactiae *(*n *= 38) including our isolates (highlighted in red) using SNP analysis. The phylogeny was rooted at midpoint. The scale bars show the number of substitutions per site. The numerical values represent 1000 bootstrap replicate values expressed as a percentage. The colors in the heat map represent the levels of identity (%) between isolates, with white indicating the lowest and green indicating the highest.**

**Additional file 7. Phylogenetic analysis of *S. pyogenes*** **(*n *= 76) including our isolates (highlighted in red) using SNP analysis. The phylogeny was rooted at midpoint. The scale bars show the number of substitutions per site. The numerical values represent 1000 bootstrap replicate values expressed as a percentage. Subtrees including the** ***S. pyogenes *sequences which showed low sequence identity with our isolates were compressed to better visualize the genetic relationships. The colors in the heat map represent the levels of identity (%) between isolates, with white indicating the lowest and green indicating the highest.**

**Additional file 8. Phylogenetic analysis of** ***S. equi*** **subsp.* zooepidemicus *(*n *= 29) from the United States including our isolates (highlighted in red) using SNP analysis. The phylogeny was rooted at midpoint. The scale bars show the number of substitutions per site. The numerical values represent 1000 bootstrap replicate values expressed as a percentage. The colors in the heat map represent the levels of identity (%) between isolates, with white indicating the lowest and green indicating the highest.**

## Data Availability

Paired-end reads of the *Streptococcus s*pp. isolates in this study were deposited in the National Center for Biotechnology Information (NCBI) under the Bioproject accession number PRJNA887842.
