# Supplementary figures and images for: Genome analysis of Streptococcus spp. isolates from animals in pre-antibiotic era with respect to antibiotic susceptibility and virulence gene profiles
Source: Vet Res. 2024 Apr 15;55:51. doi: 10.1186/s13567-024-01302-0 (PMC11017511; doi:10.1186/s13567-024-01302-0)

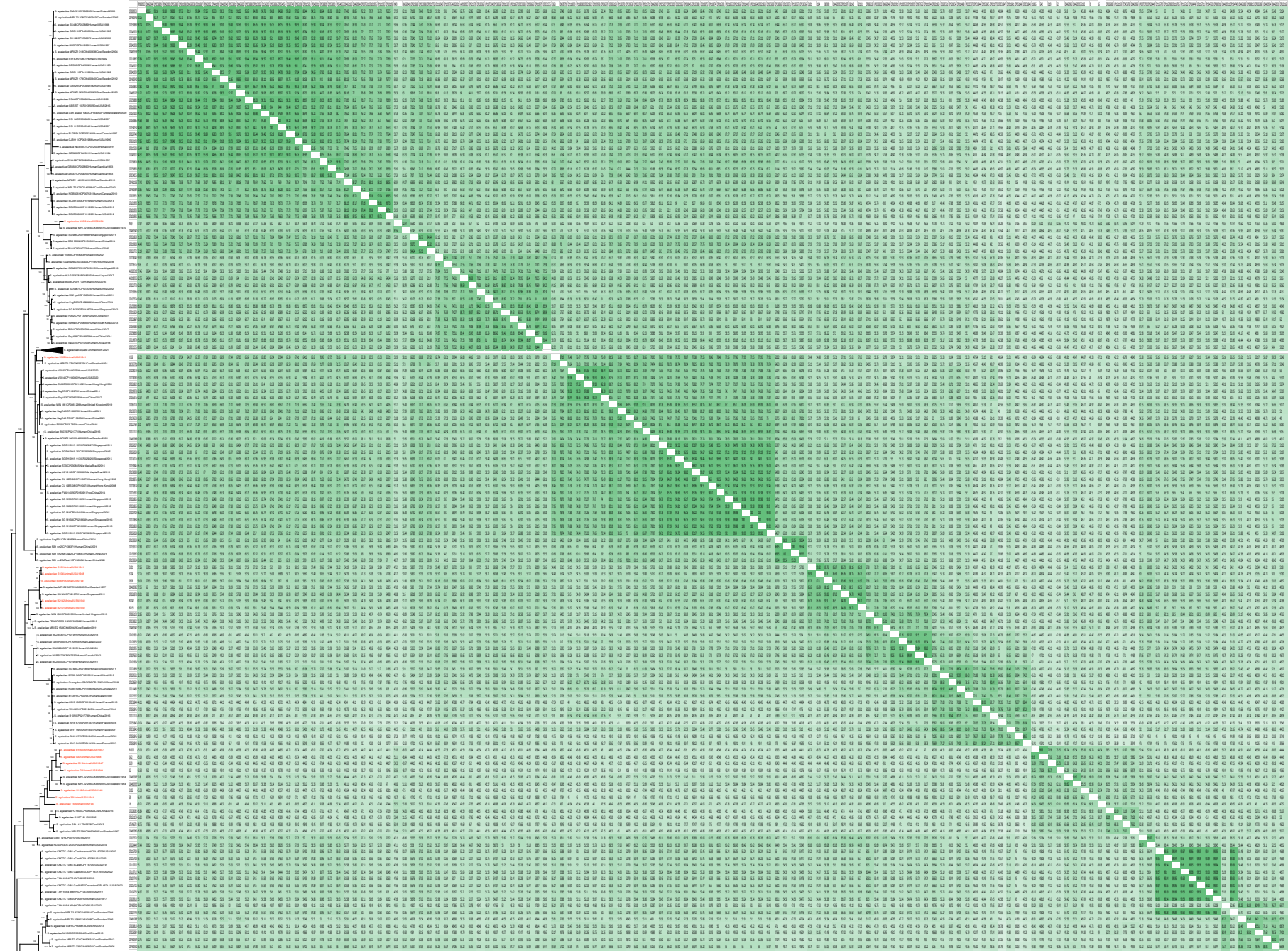

Supplement: Supplementary file 5 — Additional file 5. Phylogenetic analysis of S. agalactiae (n = 199) including our isolates (highlighted in red) using SNP analysis. The phylogeny was rooted at midpoint. The scale bars show the number of substitutions per site. The numerical values represent 1000 bootstrap replicate values expressed as a percentage. Subtrees including the S. agalactiae from aquatic animals were compressed to better visualize the genetic relationships. The colors in the heat map represent the levels of identity (%) between isolates, with white indicating the lowest and green indicating the highest. [file 13567_2024_1302_MOESM5_ESM.pdf]

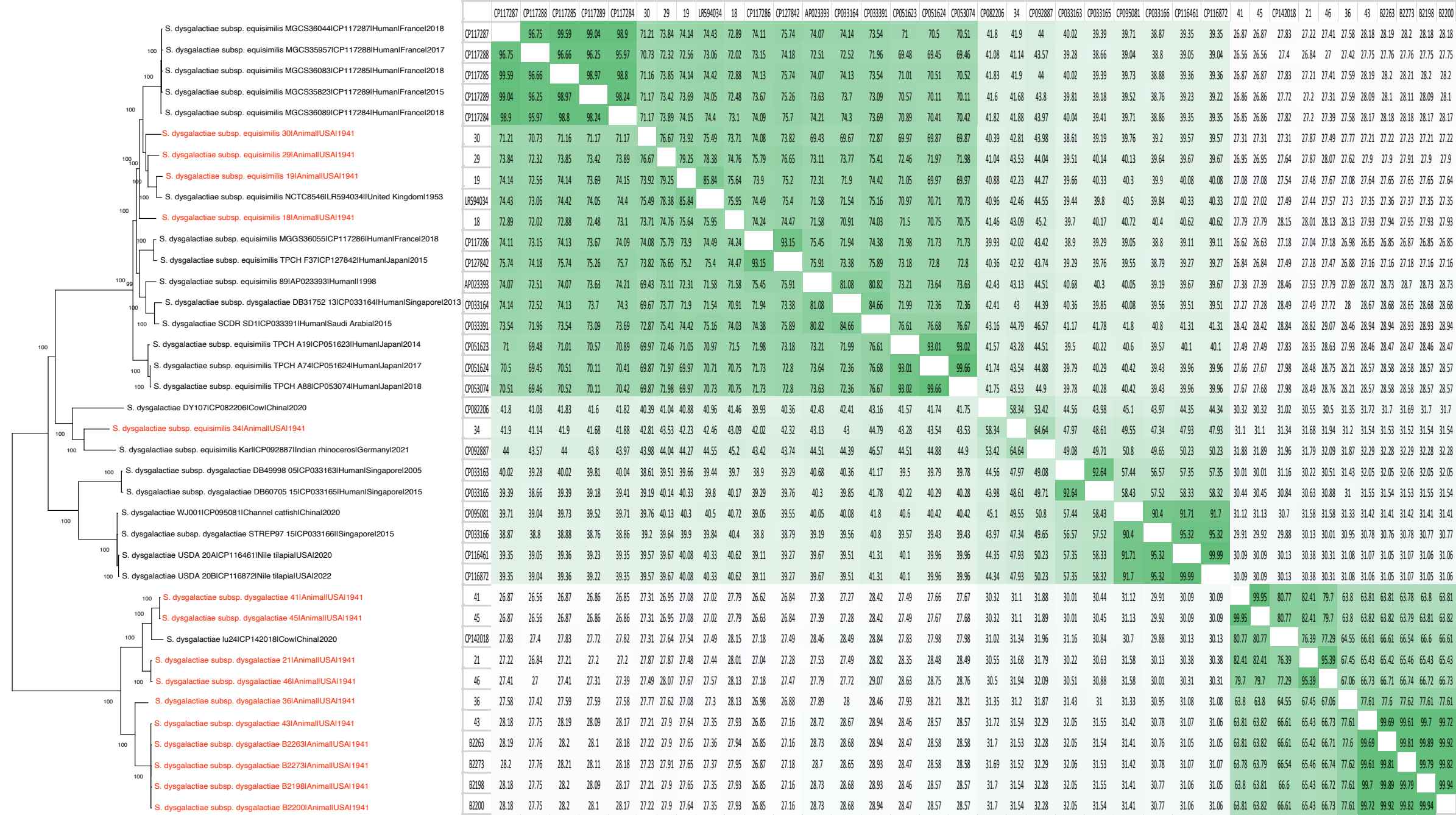

Supplement: Supplementary file 6 — Additional file 6. Phylogenetic analysis of S. dysgalactiae (n = 38) including our isolates (highlighted in red) using SNP analysis. The phylogeny was rooted at midpoint. The scale bars show the number of substitutions per site. The numerical values represent 1000 bootstrap replicate values expressed as a percentage. The colors in the heat map represent the levels of identity (%) between isolates, with white indicating the lowest and green indicating the highest. [file 13567_2024_1302_MOESM6_ESM.pdf]

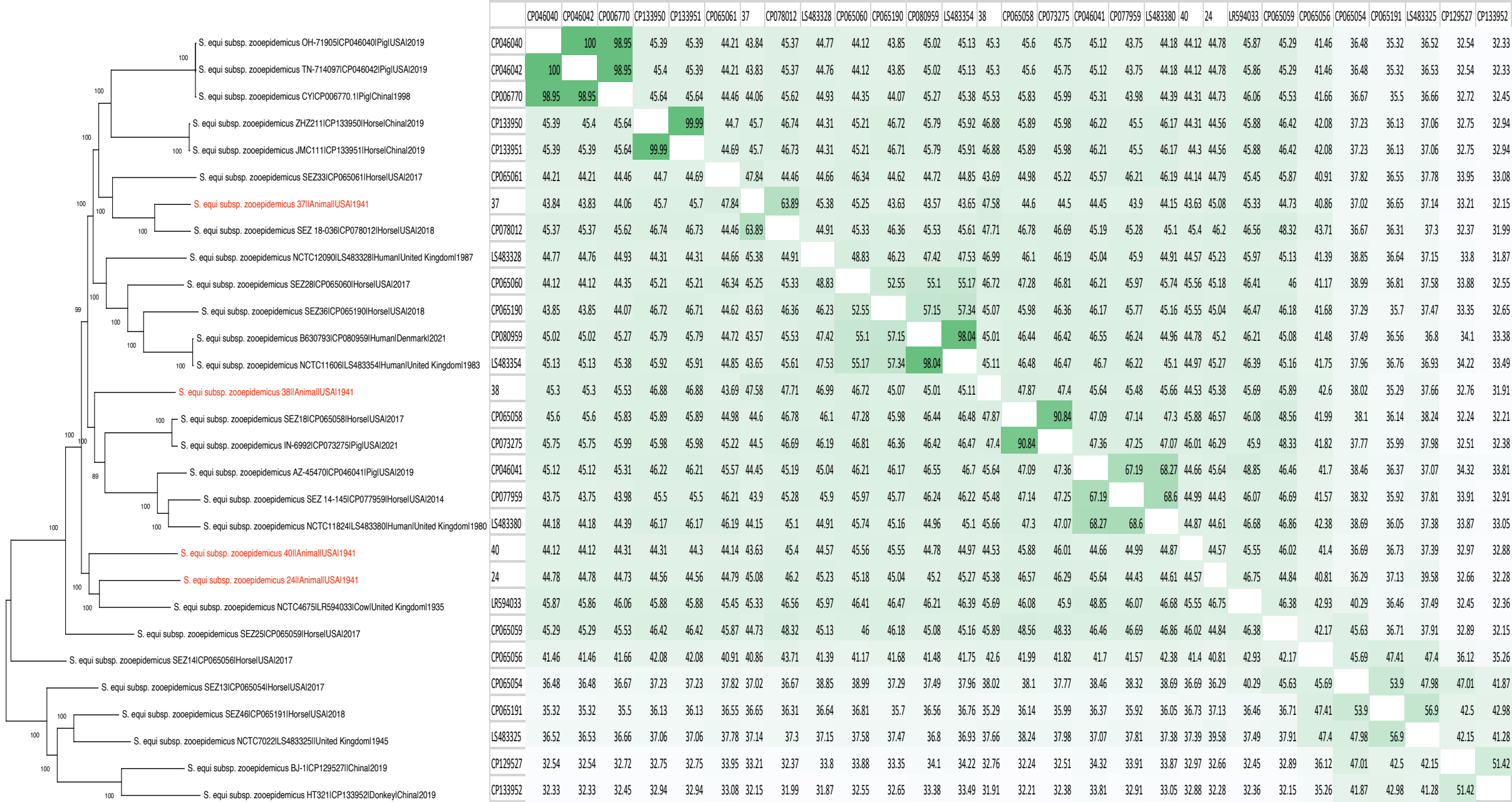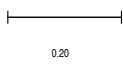

Supplement: Supplementary file 8 — Additional file 8. Phylogenetic analysis of S. equi subsp. zooepidemicus (n = 29) from the United States including our isolates (highlighted in red) using SNP analysis. The phylogeny was rooted at midpoint. The scale bars show the number of substitutions per site. The numerical values represent 1000 bootstrap replicate values expressed as a percentage. The colors in the heat map represent the levels of identity (%) between isolates, with white indicating the lowest and green indicating the highest. [file 13567_2024_1302_MOESM8_ESM.pdf]
